# Exogenous Glutathione Protects IPEC-J2 Cells against Oxidative Stress through a Mitochondrial Mechanism

**DOI:** 10.3390/molecules27082416

**Published:** 2022-04-08

**Authors:** Qiuyu Chen, Miao Yu, Zhimei Tian, Yiyan Cui, Dun Deng, Ting Rong, Zhichang Liu, Min Song, Zhenming Li, Xianyong Ma, Huijie Lu

**Affiliations:** 1 College of Animal Science & Technology, Zhongkai University of Agriculture and Engineering, Guangzhou 510225, China; chenqiuyu9713@163.com; 2 State Key Laboratory of Livestockand Poultry Breeding, Institute of Animal Science, Guangdong Academy of Agricultural Sciences, Guangzhou 510640, China; yumiao@gdaas.cn (M.Y.); tianzhimei@gdaas.cn (Z.T.); cuiyiyan@gdaas.cn (Y.C.); dengdun@gdaas.cn (D.D.); rongting@gdaas.cn (T.R.); liuzhichang@gdaas.cn (Z.L.); songmin@gdaas.cn (M.S.); lizhenming@gdaas.cn (Z.L.); 3 Key Laboratory of Animal Nutrition and Feed Science in South China, Ministry of Agriculture, Guangzhou 510640, China; 4 Guangdong Provincial Key Laboratory of Animal Breeding and Nutrition, Guangzhou 510640, China; 5 Guangdong Engineering Technology Research Center of Animal Meat Quality and Safety Control and Evaluation, Guangzhou 510640, China; 6 Qingyuan Longfa Pig Breeding Co., Ltd., Yingde 511500, China

**Keywords:** hydrogen peroxide, IPEC-J2 cells, oxidative stress, apoptosis, mitochondrial membrane potential

## Abstract

The accumulation of reactive oxygen species (ROS) triggers oxidative stress in cells by oxidizing and modifying various cellular components, preventing them from performing their inherent functions, ultimately leading to apoptosis and autophagy. Glutathione (GSH) is a ubiquitous intracellular peptide with multiple functions. In this study, a hydrogen peroxide (H${}_{2}$O${}_{2}$)-induced oxidative damage model in IPEC-J2 cells was used to investigate the cellular protection mechanism of exogenous GSH against oxidative stress. The results showed that GSH supplement improved the cell viability reduced by H${}_{2}$O${}_{2}$-induced oxidative damage model in IPEC-J2 cells in a dose-dependent manner. Moreover, supplement with GSH also attenuated the H${}_{2}$O${}_{2}$-induced MMP loss, and effectively decreased the H${}_{2}$O${}_{2}$-induced mitochondrial dysfunction by increasing the content of mtDNA and upregulating the expression TFAM. Exogenous GSH treatment significantly decreased the ROS and MDA levels, improved SOD activity in H${}_{2}$O${}_{2}$-treated cells and reduced H${}_{2}$O${}_{2}$-induced early apoptosis in IPEC-J2 cells. This study showed that exogenous GSH can protect IPEC-J2 cells against apoptosis induced by oxidative stress through mitochondrial mechanisms.

## 1. Introduction

The gastrointestinal tract, characterized by having the largest mucosal surface in the body and an ability for self-renewal, is lined with a monolayer of intestinal epithelial cells (IECs), which represents the first barrier of defense between the luminal environment and the body [1]. The intestinal epithelial barrier serves to protect the mucosal surface against injury, infection and dietary antigens, while maintaining a delicate balance between the largest population of commensal organisms and the body [1,2,3].

Due to their exposed location, IECs can be regularly injured by oxidative stress induced by endogenous- and diet-derived toxic substances and oxidants, which, in turn, stimulate IECs and immune cells to produce excessive reactive oxygen species (ROS) and pro-inflammatory mediators [4,5,6,7]. Several lines of evidence have suggested that oxidative stress impairs intestinal epithelial function, increases intestinal permeability, causes intestinal malabsorption, and is involved in the pathogenesis of a number of gastrointestinal diseases, including inflammatory bowel disease (IBD), degenerative diseases and even cancer [4,5,8,9]. Upon injury, the intestinal epithelium undergoes a wound-healing process, which is dependent on the precise balance of various cellular processes, including proliferation, differentiation, apoptosis, restitution and migration of IECs around the wound area [10]. Therefore, IECs are increasingly recognized as gatekeepers to maintain redox and immune homeostasis, by separating luminal content from underlying tissues [11].

During these cellular processes, IECs have distinct redox homeostasis features, which depend on the various changes in mitochondrial functions [11]. Mitochondrial function is the key factor in determining cell fate and coordinating cell metabolism, stress response and apoptosis. Cell-specific stress responses lead to mitochondrial dysfunction [12]. The initiating factor in the mitochondrial dysfunction could be an early alteration of the IECs redox status suggesting the importance of mitochondrial function under oxidative stress [13]. The loss of the mitochondrial membrane potential (MMP) is the key mechanism leading to the formation of ROS during stress stimulation [14]. In addition, mitochondria are also integrated into the intracellular signaling network that regulates a variety of cell processes, thereby playing a key role in the cytopathological mechanism of various diseases such as aging-related diseases, cancer, and neurodegenerative diseases [15,16,17]. Mitochondrial DNA (mtDNA) mutations and deletions can be observed in cancer, aging, and injury [17]. For example, in a study by Foote et al. [18], the analysis of multiple early and reproducible parameters of vascular aging in mice revealed that the decrease of mtDNA integrity and mitochondrial function directly promotes vascular aging. In addition, mitochondrial dysfunction and oxidative damage play an important role in several neurodegenerative diseases [17]. Studies have found that free radicals, such as NO and other oxygen-centric related species, may damage a variety of cellular macromolecules, including large molecules of the electron transport system, thereby impairing mitochondrial function [19]. The mechanism of action of many antioxidant drugs used to treat diseases is by improving mitochondrial disorders. Recently, resveratrol activates *SIRT3*, which leads to a decrease in acetylation of the mitochondrial transcription factor *TFAM*, thereby improving diabetic mitochondrial dysfunction [20].

Glutathione (L-γ-glutamyl-L-cysteyl-glycine; GSH) is a ubiquitous intracellular peptide involved in numerous cellular processes, including (1) scavenging free radicals; (2) maintaining the essential thiol status of proteins; (3) providing a reservoir for cysteine; (4) cell differentiation, proliferation and apoptosis; (5) microtubule-related processes; (6) immune function [21]. Additionally, disturbances in GSH homeostasis are involved in the etiology and progression of many diseases [21]. In particular, GSH deficiency, or a decrease in the GSH/glutathione disulfide (GSSG) ratio, leads to an increased susceptibility to oxidative stress implicated in the progression of cancer, and elevated GSH levels increase the antioxidant capacity and the resistance to oxidative stress as observed in many cancer cells [22].

As with most tissues, IECs contain many GSH-dependent systems in all segments of the gastrointestinal tract that function to preserve cellular redox homeostasis and intestinal cell integrity [23,24]. In IECs, γ-glutamyl transferase (γ-GT) and dipeptidase (DP) catalyze the hydrolysis of extracellular GSH to its constituent amino acids, glutamic acid, cysteine and glycine. It is also possible to import complete GSH from the cavity through a specific plasma membrane transporter [1]. GSH is distributed among organelles, such as mitochondria, endoplasmic reticulum and nucleus, in an independent manner, which plays an important role of antioxidant and constitutes the main antioxidant defense system in cells [21,25,26,27,28,29]. Previously, it has been demonstrated that GSH deficiency causes intestinal epithelial degeneration, while exogenously administered GSH reverses jejunal and colonic degeneration [1,30]. Therefore, the exogenously administered GSH is considered to be a promising strategy for the treatment of intestinal epithelial injury.

To measure subcellular protective mechanisms of extracellular GSH, we focused on mitochondrial function in porcine IPEC-J2 intestinal epithelial cells. Hydrogen peroxide (H${}_{2}$O${}_{2}$) treatment was used to induce cell injury and establish a cell injury model of oxidative stress in IPEC-J2. The use of H${}_{2}$O${}_{2}$ as insult agent is a widespread method used to mimic the pro-oxidative environment that characterizes degenerative disease [31]. Based on the available evidence, we hypothesize that GSH inhibits the H${}_{2}$O${}_{2}$-induced oxidative damage and promotes mitochondrial function, thereby improving the intestinal epithelial antioxidant defense system. In this study, the MMP, mtDNA content, ROS activity, cell viability and apoptosis were measured to verify our hypothesis. This study highlights the cytoprotective effects of exogenously administered GSH against the mitochondrial dysfunction in oxidative stress-impaired IECs. We anticipate that this study will provide an easy approach for improving intestinal health.

## 2. Methods

### 2.1. Cell Culture

Porcine IPEC-J2 intestinal cells, are porcine intestinal columnar epithelial cells that were isolated from neonatal piglet mid-jejunum, and were kindly provided by Dr Yin Yulong’s laboratory (Institute of Subtropical Agriculture, Chinese Academy of Sciences, Changsha, China). Cells were grown in Dulbecco’s modified Eagle’s medium (DMEM) base supplemented with 10% FBS (Sigma, St. Louis, MO, USA), and 1% penicillin-streptomycin (100 U/mL). The cells were cultured in an incubator at 37 ℃ with a humidified atmosphere of 5% CO${}_{2}$ in air. The culture medium was changed three times a week, according to standard cell culture protocols.

### 2.2. Reagents

The Annexin V/Propidium Iodide (PI) Apoptosis Detection kit, Reactive Oxygen Species Assay Kit, Total Superoxide Dismutase Assay Kit with WST-8, Lipid Peroxidation MDA Assay Kit and MMP assay kit with JC-1 were purchased from Beyotime Institute of Biotechnology (Shanghai, China). The Cell Counting Kit-8 (CCK-8) was purchased from Dojindo Molecular Technologies, Inc. (Kumamoto, Japan). Other materials and chemicals were purchased from commercial sources.

### 2.3. CCK-8 Cell Proliferation Assay

Cell proliferation was measured using a CCK-8 assay, based on the enzymatic reduction of the water-soluble tetrazolium-8 (WST-8) salt (2-(2-methoxy-4-nitrophenyl)-3-(4-nitrophenyl)-5-(2,4-disulfophenyl)-2H-tetrazolium) in living cells and the production of a color change proportional to cell viability. Briefly, 5 × 10${}^{3}$ cells suspended in 150 μL of complete medium were seeded in each well of a 96-well culture plate (Corning, #3872; Corning Inc., Corning, NY, USA) and incubated for 24 h at 37 °C in an incubator with a humidified atmosphere of 5% CO${}_{2}$ in air. The WST-8 cell proliferation reagent (50 μL) was added, and plate was incubated for another 4 h at 5% CO${}_{2}$ and 37 °C. The negative control consisted of WST-8 reagent and complete medium with no cells. The absorbance was measured at 450 nm using a spectrophotometer and the optical density (OD) was calculated as follows: OD = OD${}_{Sample}$− OD${}_{Control}$. Each experiment was performed in sextuplicate wells and repeated three times.

### 2.4. Cell Migration Assay

Cells were allowed to grow to just more than 90% confluent with no gaps between cells, except for two separate wounds through the cell monolayer with a sterile 200 μL pipet tip. We tried to maintain consistent widths and straight edges for all the experiments. After scratching, cells were pretreated with or without H${}_{2}$O${}_{2}$ (500 μM) for 24 h. Afterwards, the culture medium was removed and replaced with fresh medium with or without GSH (1.6 mM) and incubated for another 24 h. The width of the migrated scratch was quantified at three time points.

### 2.5. Measurement of Mitochondrial Membrane Potential (MMP)

JC-1 probe was employed to measure mitochondrial depolarization in IPEC-J2. Briefly, Cells cultured in six-well plates after indicated treatments were incubated with an equal volume of JC-1 staining solution (5 μg/mL) at 37 °C for 20 min and rinsed twice with PBS. Mitochondrial membrane potentials were monitored by determining the relative amounts of dual emissions from mitochondrial JC-1 monomers or aggregates using the Nikon Opera system (Nikon Corporation, Tokyo, Japan) under Argon-ion 488 nm laser excitation. Mitochondrial depolarization is indicated by an increase in the green/red fluorescence intensity ratio.

### 2.6. RNA Isolation and Quantitative Real-Time PCR (qPCR)

Total RNA was isolated from IPEC-J2 cells using TRIzol (Invitrogen, Carlsbad, CA, USA), and quantified based on the absorbance at 260 nm using NanoDrop 2000 (Thermo Scientific, Waltham, MA, USA). Reverse transcription was performed on 1 μg total RNA using MMLV reverse transcriptase (Invitrogen) according to manufacturer’s instructions, and the cDNA product was used as template for PCR analysis. The primers of the target genes, namely *TFAM*, *TFB1M* and *TFB2M*, and house-keeping genes, namely *B2M*, *HPRT1* and *RPL4*, are shown in Table 1. A reliable standard has been established for the standardization of qPCR. The geNorm method was used to sort the four potential reference genes by stability and determine the most stable reference genes from the set of tested candidate reference genes [32].

### 2.7. DNA Extraction and Quantification of mtDNA Copy Number

DNA was isolated from all tissues using the Animal Genomic DNA Quick Extraction Kit for PCR Analysis (D0065M; Beyotime Institute of Biotechnology) according to the manufacturer protocol. The nuclear gene glucagon (GCG) was used as the single-copy reference gene. Relative amounts of mtDNA and nucDNA(GCG) were determined by qPCR. To determine the mitochondrial DNA content, each sample was normalized to the expression level of the single-copy reference gene GCG and the value expressed as the ratio to the control group [33].

### 2.8. Reactive Oxygen Species (ROS) Detection

To measure ROS levels, IPEC-J2 cells were grown to confluence in a 96-well plate in the presence or absence of GSH and/or H${}_{2}$O${}_{2}$, washed once with the best reduced serum medium (Invitrogen, San Diego, CA, USA), and incubated with 20 μM of the cell-permeable fluorescent dye 2${}^{\prime }$,7${}^{\prime }$-dichlorofluorescein diacetate (DCFH-DA) (Beyotime Institute of Biotechnology) at 37 °C for 30 min. After incubation with the fluorescent probe, the Nikon Opera system (Nikon Corporation, Tokyo, Japan) was used to immediately image the fluorescent DCFH probe at 37 °C, 5% CO${}_{2}$, and 60% humidity lifetime imaging conditions. The total fluorescence intensity of DCFH-DA in cells was measured using a Spectra Max M2 plate reader (Molecular Devices, San Jose, CA, USA) at excitation and emission wavelengths of 485 and 525 nm, respectively.

### 2.9. Evaluation of Malondialdehyde (MDA) and Superoxide Dismutase (SOD) Levels

As described previously, the cells were seeded in 6-well plates, treated with H${}_{2}$O${}_{2}$ (500 μM) and GSH (1.6 mM). After the treatment, the lipid peroxidation MDA Assay Kit (S0131M; Beyotime Institute of Biotechnology) and total SOD Assay Kit with WST-8 (S0101M; Beyotime Institute of Biotechnology) were used to determined MDA levels and SOD activity, respectively, in cell homogenates according to the manufacturer’s instructions

### 2.10. Cell Apoptosis Assay

The BD annexin V(PE)/7-AAD Apoptosis detection kit (556547; BD Biosciences, San Jose, CA, USA) was used to determine cell apoptosis rate according to the manufacturer’s instructions. The cells were seeded at a density of 1 × 10${}^{6}$ cells/well in 6-well plates and allowed to grow to 70 % confluence. After digestion with trypsin and washing twice with PBS, the cells were labeled with 5 μL Annexin V-fluorescein isothiocyanate (PE) and 5 µL 7-AAD for 5 min each, at room temperature in the dark. The cell suspensions were analyzed by flow cytometry using the Cell Quest version 3.3 software (BD Biosciences).

### 2.11. Statistical Analysis

All data are presented as the mean ± standard deviation of at least three independent experiments. Statistical analyses were performed Using GraphPad Prism version 8.0.0 for Windows (GraphPad Software, San Diego, CA, USA), perform Dunnett’s multiple comparison test after one-way analysis of variance. Multiple comparisons were performed using one-way analysis of variance (ANOVA) test with Schaffer’s post hoc test when the distribution was normal, or using the non-parametric Kruskal–Wallis test with a Dunn’s post hoc test when the distribution was not normal. A *p* < 0.05 (two-tailed) was considered to indicate a statistically significant difference.

## 3. Results

### 3.1. Exogenous GSH Improves IPEC-J2 Viability after H${}_{2}$O${}_{2}$ Exposure

**Figure 1:**
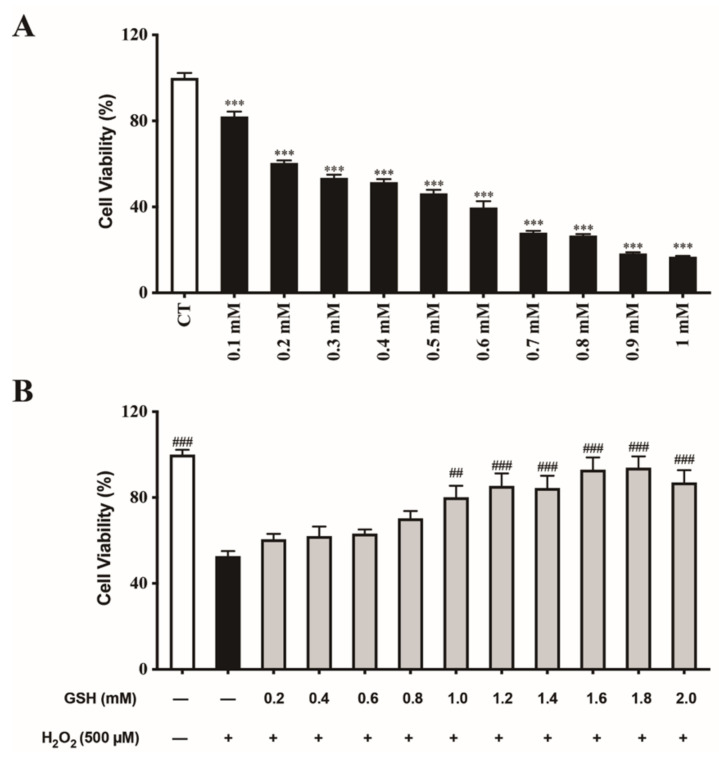
Protective effect of GSH on H${}_{2}$O${}_{2}$-induced cytotoxicity in IPEC-J2 cells. (**A**) Cells were seeded in 96-well plates (5 × 10${}^{3}$ cells per well) and treated with H${}_{2}$O${}_{2}$ (500 μM) for 24 h. The cell viability was determined by the CCK-8 assay. Values are presented as the mean ± SEM (*n* = 6). *** *p* < 0.001 vs. control (CT) group. (**B**) Cells were pretreated with H${}_{2}$O${}_{2}$ (500 μM) for 24 h. Subsequently, the culture medium was removed from the wells and replaced with fresh medium supplemented without or with GSH at the indicated concentration and the plate was incubated for another 24 h. Subsequently, the medium was removed, and cell viability was measured using the CCK-8 assay. Values are presented as the mean ± SEM (*n* = 6). ## *p* < 0.01, ### *p* < 0.001 vs. H${}_{2}$O${}_{2}$-treated group.

Cell viability was measured after exposure to H${}_{2}$O${}_{2}$ and treatment with or without GSH at different concentrations. The results revealed that H${}_{2}$O${}_{2}$ inhibited the viability of IPEC-J2 cells in a dose-dependent manner (Figure 1A). Compared with the control group, exposure to 500 μM for 24 h resulted in about 50% decrease in the rate of cell viability. Therefore, the treatment with a concentration of 500 μM H${}_{2}$O${}_{2}$ for 24 h was chosen as the model condition of oxidative stress in IPEC-J2 for subsequent studies. The results also demonstrated that GSH treatment attenuated H${}_{2}$O${}_{2}$-induced reduction of cell viability in a dose-dependent manner (Figure 1B).

### 3.2. Exogenous GSH Attenuated H${}_{2}$O${}_{2}$-Inhibited Migration of IPEC-J2 Cells

**Figure 2:**
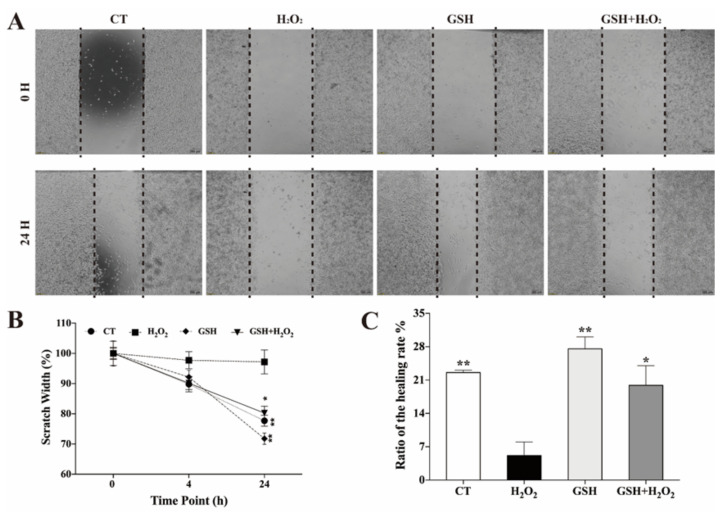
Effect of GSH on the cell migration capacity of IPEC-J2. (**A**) Representative image of wound-healing assay. The dashed lines indicate wound edges. After scratching, cells were pretreated with or without H${}_{2}$O${}_{2}$ (500 μM) for 24 h. Afterwards, the culture medium was removed, replaced with fresh medium with or without GSH (1.6 mM) and incubated for another 24 h. Scale bar: 200 μm. (**B**) Quantification of migrated scratch width at different time point. Values are presented as the mean ± SD (*n* = 3). * *p* < 0.05 vs. H${}_{2}$O${}_{2}$-treated group at the same time point. (**C**) Quantification of the healing rate. The healing or closure rate is expressed as a ratio of the migration distance (after 24 h) compared with the distance immediately after scratching. Values are presented as the mean ± SD (*n* = 3). ** *p* < 0.01 compared with the H${}_{2}$O${}_{2}$-exposed IPEC-J2.

In the wound-healing assay, GSH was able to increase the basal and H${}_{2}$O${}_{2}$-inhibited migration of IPEC-J2 cells after 24 h (Figure 2). After scratching, IPEC-J2 cells generally began to migrate into the vacated space (Figure 2A). As a result of H${}_{2}$O${}_{2}$-induced oxidative stress, the migration capacity of IPEC-J2 cells was significantly hindered, and exogenous GSH rescued the H${}_{2}$O${}_{2}$induced impairment of migration (Figure 2B,C). The healing rate of IPEC-J2 treated with H${}_{2}$O${}_{2}$ was much lower than that of the control and GSH treated groups (*p* < 0.01).

### 3.3. Exogenous GSH Rescues the H${}_{2}$O${}_{2}$-Impaired Mitochondrial Function

**Figure 3:**
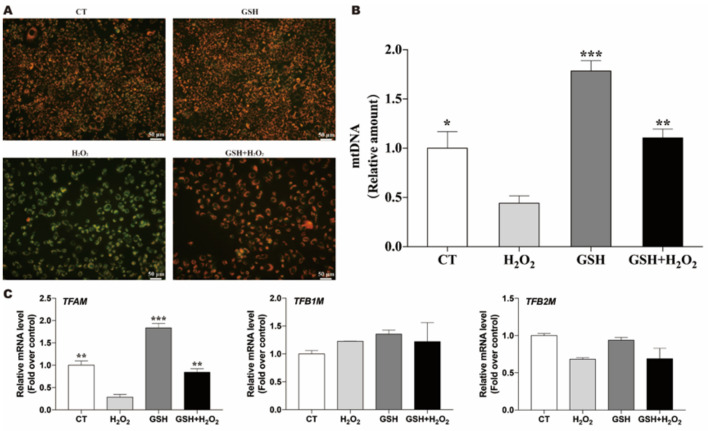
GSH rescues the H${}_{2}$O${}_{2}$-impaired mitochondrial function. Cells were pretreated with or without H${}_{2}$O${}_{2}$(500 μM) for 24 h. Afterwards, the culture medium was removed replaced with fresh medium with or without GSH (1.6 mM) and incubated for another 24 h. (**A**) MMP was determined by measuring the fluorescence intensity of JC-1 monomers and aggregates. (**B**) The mitochondrial copy number was determined to investigate the mitochondrial biogenesis. Data are presented as the mean ± SEM of three independent experiments. * *p* < 0.05 compared with the H${}_{2}$O${}_{2}$-exposed IPEC-J2. (**C**) Effects of exogenous GSH on the expression of mitochondrial transcription factors. ** *p* < 0.01 compared with the H${}_{2}$O${}_{2}$-exposed IPEC-J2. *** *p* < 0.001 compared with the H${}_{2}$O${}_{2}$-exposed IPEC-J2.

In this study, the results demonstrated that supplementation with exogenous GSH rescued the H${}_{2}$O${}_{2}$-induced MMP depolarization (Figure 3A). To further evaluate the protective effects of GSH on mitochondrial function, mtDNA content and the expression of three mitochondrial transcription factors (*TFAM*, *TFB1M* and *TFB2M*) were investigated after exposure to H${}_{2}$O${}_{2}$ in IPEC-J2 cells. MtDNA content was reduced after the exposure to H${}_{2}$O${}_{2}$ in IPEC-J2 cells. GSH effectively rescued H${}_{2}$O${}_{2}$-induced mitochondrial dysfunction by increasing mtDNA content and upregulating *TFAM* expression (Figure 3B,C).

### 3.4. GSH Reduces H${}_{2}$O${}_{2}$-Induced ROS and Oxidative Stress

**Figure 4:**
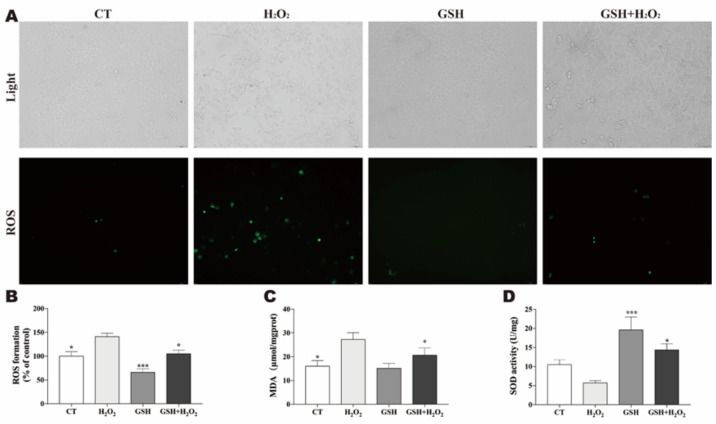
GSH attenuates H${}_{2}$O${}_{2}$-induced oxidative stress. Cells were pretreated with or without H${}_{2}$O${}_{2}$ (500 μM) for 24 h. Afterwards, the culture medium was removed replaced with fresh medium with or without GSH (1.6 mM) and incubated for another 24 h. (**A**) After treatment, the normal image of the cells in the same field of view and the fluorescence image showing the level of ROS were captured. (**B**) ROS generation determined by measuring the fluorescence intensity of an oxidation-sensitive fluorescein DCFH-DA. Data represent means ± S.E.M. (*n* = 6) and differences between mean values were assessed by one-way ANOVA. *** *p* < 0.001 indicate the significant difference compared with H${}_{2}$O${}_{2}$-treated group. (**C**) Quantification of MDA generation. (**D**) Quantification of SOD activity. Values are presented as the mean ± SEM (*n* = 3). * *p* < 0.05 compared with the H${}_{2}$O${}_{2}$-exposed IPEC-J2. *** *p* < 0.001 compared with the H${}_{2}$O${}_{2}$-exposed IPEC-J2.

Our results suggested that treatment of IPEC-J2 cells with H${}_{2}$O${}_{2}$ alone increased the intracellular ROS production and the level of MDA, and inhibited SOD activity. In contrast, treatment with exogenous GSH noticeably reversed the H${}_{2}$O${}_{2}$-elevated ROS and MDA levels, and restored H${}_{2}$O${}_{2}$-impaired SOD activity (Figure 4).

### 3.5. Exogenous GSH Reduces H${}_{2}$O${}_{2}$-Induced Cell Apoptosis

**Figure 5:**
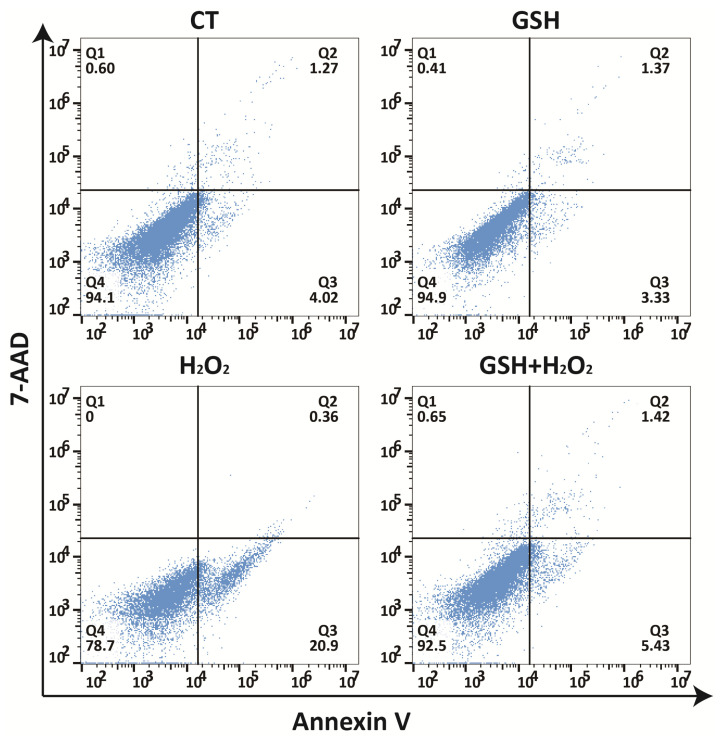
Protective effects of GSH against H${}_{2}$O${}_{2}$-induced apoptosis in IPEC-J2 cells. Cells were pretreated with or without H${}_{2}$O${}_{2}$ (500 μM) for 24 h. Afterwards, the culture medium was removed replaced with fresh medium with or without GSH (1.6 mM) and incubated for another 24 h, and cell distribution was analyzed using Annexin V-PE binding and 7-AAD uptake. The PE and 7-AAD fluorescence intensity were measured by flow cytometry using the FL-2 and FL-3 filters, respectively. Q4, living cells (Annexin V-/7-AAD-); Q3, early apoptotic/primary apoptotic cells (Annexin V+/7-AAD-); Q2, late apoptotic/necrotic cells (Annexin V+/7-AAD+).

After the treatment with or without GSH and H${}_{2}$O${}_{2}$, IPEC-J2 cells were double stained with phycoerythrin (PE)-labeled Annexin V (Annexin V-PE) and 7-AAD to analyze the apoptotic cells by flow cytometry. The results revealed that H${}_{2}$O${}_{2}$ exposure clearly induced early apoptosis, and GSH inhibited H${}_{2}$O${}_{2}$-induced early apoptosis in IPEC-J2 cells (Figure 5).

## 4. Discussion

IECs have the physiological functions of digestion, absorption and immunity, and are extremely susceptible to oxidative damage caused by exogenous ROS [34]. In this study, we found that exogenous GSH can restore the cell viability of H${}_{2}$O${}_{2}$-impaired IPEC-J2 cells in a dose-dependent manner. The oxidative damage model with cell survival rate reduced by about 50% can be restored to near normal cell viability by supplementing with 1.6 mM GSH. Therefore, we chose to pretreat the IPEC-J2 cells with H${}_{2}$O${}_{2}$ (500 μM) for 24 h, and then supplement with GSH (1.6 mM) for another 24 h as an exploratory model to investigate how exogenous GSH protects IPEC-J2 cells from oxidative damage.

Collective cell migration is a hallmark of wound repair, cancer invasion and metastasis, immune response, angiogenesis and embryonic morphogenesis [35]. As porcine epithelial cells, after being injured, IPEC-J2 cells will migrate from the edge of the wound to the wound to restore the integrity of the skin [13]. We speculate that after oxidative damage, cells will lose the corresponding protective migration function, and such function can be restored in damaged cells by exogenous addition of GSH. Therefore, the “in vitro scratch test” was used to study the effect of the treatment with GSH on the migration capacity of IPEC-J2 cells exposed to H${}_{2}$O${}_{2}$. After the scratching, there were significant differences in the migration capacity between the cells treated with or without H${}_{2}$O${}_{2}$ and GSH. The healing rate of the H${}_{2}$O${}_{2}$-treated group was significantly slower than that of the other treatment groups, and the corresponding protective migration capacity was lost. Compared with the control group, the healing rate of the experimental group treated only with GSH tended to increase. In fact, the healing rate of the experimental group pretreated with H${}_{2}$O${}_{2}$ was basically restored to the level of the control group after GSH was added to the cells. These results confirmed our hypothesis that exogenous GSH can effectively restore the protective migration ability of cells lost due to oxidative damage.

Mitochondria are both the main producers and targets of ROS. Under oxidative damage conditions, the production of ROS may be accompanied by a decrease in the MMP. Moreover, mitochondrial transcription factor A (*TFAM*) is an mtDNA binding protein and transcription/replication factor that plays an essential role in the maintenance of mtDNA and mitochondrial homeostasis, usually related to the copy number of mtDNA [26]. Overexpression of TFAM will increase the copy number of mtDNA [36]. The continuous replication state of mtDNA and the existence of nucleoid structure make mitochondria vulnerable to oxidative damage and mutation [36]. *TFB1M* and *TFB2M* are protein-coding genes, and their related pathways include mitochondrial gene expression and the biogenesis and maintenance of organelles [37]. Among the battery of antioxidants that protect mitochondria from ROS, GSH is thought to be essential for the antioxidant function of this organelle [25]. However, mitochondria cannot synthesize their own GSH de novo, and thus rely on an efficient transport of GSH from the cytosol to maintain their redox status [25]. Therefore, the present study aimed to test whether addition of exogenous GSH can alleviate the oxidative stress-induced mitochondrial dysfunction. In Figure 3, we found that exogenous GSH supplementation prevented H${}_{2}$O${}_{2}$-impaired MMP and mitochondrial biogenesis, which might be relate with the modulation of *TFAM*.

In the physiological state, ROS acts as a signal molecule to support normal physiological activities [38]. Once oxidative damage occurs, an excessive amount of ROS can be produced, which is more likely to induce apoptosis [39]. Accordingly, we also paid attention to the changes in ROS levels as well as the changes in the MDA content and SOD activity as indicators of changes in oxidative damage. Our results revealed that exogenous GSH significantly repressed the H${}_{2}$O${}_{2}$-induced increase in ROS and MDA formation, and rescued the activity of SOD. In addition, the loss of MMP and increased oxidative damage represent a key step of apoptosis [14]. The results of this study showed that H${}_{2}$O${}_{2}$ exposure significantly induced early apoptosis, and GSH reduced the H${}_{2}$O${}_{2}$-induced early apoptosis in IPEC-J2 cells. Thus, the study suggests that GSH can significantly prevent early apoptosis caused by oxidative damage.

**Figure 6:**
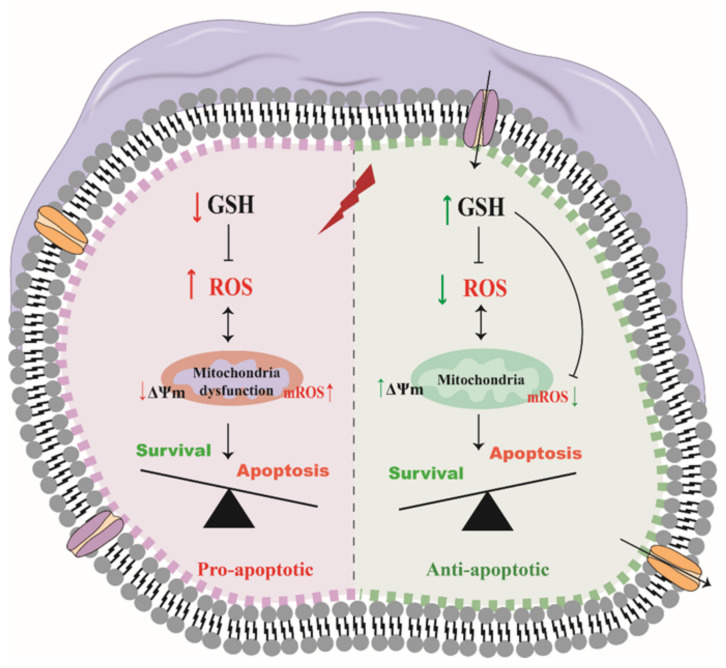
Mechanisms for ROS formation and GSH depletion/supplementation during oxidative stress. Oxidative stress led to increased mROS generation, which followed by a concomitant decrease in mitochondrial membrane potential (MMP). ROS accumulation and GSH depletion might further contribute to ROS-mediated apoptosis by mitochondrial dysfunction. Extracellular GSH alleviates oxidative stress via reducing mROS output.

Taken together, under normal conditions, the GSH and ROS in the IPEC-J2 cells undergo redox to maintain a relatively balanced state. In contract, under H${}_{2}$O${}_{2}$-induced oxidative stress conditions, it results in significantly increases the level of intracellular ROS and breaks the balance of intracellular GSH and ROS. The integrity of mitochondria is impaired, and the mitochondria cannot normally transcribe and translate the corresponding mtDNA, which leads to early cell apoptosis. The addition of exogenous GSH improves this situation by maintaining the balance between GSH and ROS, restoring the normal function of mitochondria, and inhibiting early apoptosis in cells (Figure 6).

## Figures and Tables

**Table 1:** Primers used for real-time-PCR-specific amplification.

Gene	Sequence (5*’* to -3*’*)
ND1	GACTAAACCAAACCCAACT GGGATAGGGATAAAGTTGT
GCG	GAATCAACACCATCGGTCAAAT CTCCACCCATAGAATGCCCAGT
TFAM	GACTACTGCGTCTGCACCTT GCAACTCTTCAGACCTCGCT
TFB1M	CCGTTGCCCACAATTCGAGA TCAACCACCAGAAGTTCAGCA
TFB2M	TCCTGCATACGGAGCCTTG AATGGTCTACCAGCATGGCG
B2M	TATCTGGGTTCCATCCG AACTATCTTGGGCTTATCG
HPRT1	ATCATTATGCCGAGGATTTGGA CCTCCCATCTCTTTCATCACATCT
RPL4	GCTCTATGGCACTTGGCGT GCGGAGGGCTCTTTGGAT

## Data Availability

Data are contained within the article.
